# Epidemiological Challenges and Health Disparities in the Eastern Mediterranean Region: A Region in Crisis

**DOI:** 10.5334/aogh.5199

**Published:** 2026-07-01

**Authors:** Saima Afaq, Anum Fatima Qazi, Ariya, Zala

**Affiliations:** 1 Institute of Public Health and Social Sciences, Khyber Medical University (KMU), Peshawar, Pakistan; 2 Department of Health Sciences, University of York, Heslington, York, United Kingdom; 3 Department of General Medicine, Khyber Medical College, Peshawar, Pakistan

**Keywords:** Eastern Mediterranean region (EMR), non-communicable diseases (NCDs), communicable diseases (CDs)

## Abstract

*Background:* The World Health Organization’s (WHO) Eastern Mediterranean Region (EMR) is marked by socioeconomic diversity, political instability, displacement, and environmental risks. With nearly 745 million people across 22 countries, the region faces a complex “triple burden” of non-communicable diseases (NCDs), communicable diseases (CDs), and injuries.

*Objective:* This paper provides an updated overview of the epidemiological landscape of the EMR by linking current disease burdens with socioeconomic, behavioural, environmental, and health system determinants. It highlights regional disparities and identifies priority areas for health system strengthening.

*Methods:* A descriptive narrative review of secondary data and published literature was conducted. Publicly available datasets, institutional reports, and peer-reviewed sources were purposively selected from WHO, WHO-Regional Office for the Eastern Mediterranean (WHO EMRO), the World Bank, Global Burden of Disease/Institute for Health Metrics and Evaluation (GBD/IHME), and related literature. The review focused mainly on data published between 2015 and 2022 and included indicators on demographics, mortality, disease burden, risk factors, health system capacity, and health financing across EMR countries. Findings were organised narratively to describe regional patterns, disparities, and policy-relevant gaps. No systematic review, meta-analysis, or inferential statistical modelling was undertaken.

*Findings:* The review shows substantial health inequalities across income groups. Life expectancy reaches 79.7 years in high-income countries but only 63.7 years in low-income countries. NCDs are now the leading cause of death, driven by obesity, hypertension, tobacco use, and other behavioural risks. Meanwhile, CDs and injury-related mortality remain major challenges, particularly among young people and populations affected by conflict and displacement. Health systems are constrained by fragility, high out-of-pocket expenditure, and a projected workforce shortfall of 2.1 million by 2030.

*Conclusion:* Addressing the EMR’s epidemiological challenges requires stronger primary healthcare and progress towards universal health coverage through integrated strategies targeting social determinants, sustainable financing, and improved surveillance.

## Introduction

The World Health Organization’s (WHO’s) Eastern Mediterranean Region (EMR) comprises 22 countries: Afghanistan, Bahrain, Djibouti, Egypt, Iran, Iraq, Jordan, Kuwait, Lebanon, Libya, Morocco, Oman, Pakistan, Palestine (including East Jerusalem), Qatar, Saudi Arabia, Somalia, Sudan, Syria, Tunisia, the United Arab Emirates and Yemen [1]. With a combined population of nearly 745 million people, the region’s demographics are significantly influenced by its most populous nations: Pakistan, Egypt and Iran, which together account for over half of the total population [2]. The EMR is diverse not only in terms of culture and geography, but also in economic status. The region faces some of the world’s most severe humanitarian and health crises as it hosts over 58% of the global population of refugees and internally displaced persons due to conflicts and instability in several member countries [3]. This unique context creates a challenging public health environment. Populations across the region face a ‘triple burden’ of non-communicable diseases (NCDs), communicable diseases (CDs), and injuries. These challenges are further exacerbated by humanitarian crises and political instability. Ongoing armed conflicts, population displacement, climate-related risks and economic instability continue to intensify existing health inequalities and place additional strain on healthcare systems across the region.

A foundational analysis by the Global Burden of Disease (GBD) 2015 Collaborators detailed the region’s burden of diseases, injuries and risk factors from 1990 to 2015 from the GBD 2015 study, highlighting a significant epidemiological transition towards NCDs [4]. While this work provided a critical baseline, the subsequent decade has seen the EMR’s health landscape further reshaped by new and escalating challenges, including the COVID-19 pandemic, worsening climate change impacts and protracted conflicts. There is a need for an updated analysis that not only presents the epidemiological data but also integrates it within the broader context of health systems and their underlying determinants. Despite the growing burden of disease in the EMR, comprehensive and updated regional syntheses integrating epidemiological trends, health determinants and health system capacities remain limited. This review aims to address this gap by providing a consolidated overview of the region’s evolving public health challenges. This paper aims to fill that gap by synthesising the most recent data to provide an overview that connects the disease burden with the socioeconomic, behavioural, and environmental factors shaping it.

## Methods

This paper was designed as a descriptive narrative review of secondary data and published literature on epidemiological challenges and health disparities in the WHO EMR.

Sources were selected purposively from established and publicly available international datasets, institutional reports and peer-reviewed literature. Priority was given to sources that provide regional or country-level data for EMR countries, including the WHO, WHO Regional Office for the Eastern Mediterranean (WHO EMRO), World Bank and GBD studies, Institute for Health Metrics and Evaluation (IHME), and relevant peer-reviewed publications. Where possible, the most recent available data between 2015 and 2022 were used, although earlier landmark publications were included where they provided important regional context or baseline comparisons.

Indicators were selected based on their relevance to the manuscript’s main themes: population and socioeconomic profile, mortality and health outcomes, CDs, NCDs, injuries, behavioural and environmental risk factors, health workforce, service capacity and health financing. Data were included when they were available for multiple EMR countries or were considered important for understanding regional disparities, particularly between high-income, middle-income, low-income and conflict-affected settings.

The findings were organised descriptively and narratively around the triple burden of disease, underlying determinants of health, health system constraints and opportunities for regional health governance.

## Socio-Demographic and Health System Profile

According to the World Bank classification, the countries are divided into four income groups: low-income, lower-middle-income, upper-middle-income and high-income countries [2]. This economic variation contributes to varying health challenges and resource allocations across the region. This economic stratification, detailed in Table 1, directly correlates with vast disparities in health financing, resource allocation and human development outcomes across the region. For instance, per capita health expenditure ranges from over $4000 in the United Arab Emirates to just over $100 in Yemen, illustrating the huge gap in available resources.

**Table 1:** Demographic, economic and health profiles of EMR countries.

CATEGORY	POPULATION GROWTH			ECONOMY		HEALTH FINANCING			DEVELOPMENT
INDICATORS	TOTAL (MILLION)	ANNUAL GROWTH (%)	> 65 YEARS (%TOTAL)	GNI/ CAPITA	ANNUAL GROWTH GDP (%)	TOTAL EXPENDITURE ON HEALTH (%GDP)	GOVERNMENT HEALTH EXPENDITURE (% TOTAL) (2021)	PER CAPITA TOTAL EXPENDITURE ON HEALTH (USD)	HDI (INDEX, RANK) (2022)
**High-income countries**									
**Bahrain**	1,485,509	0.9	4	28,280	2.5	4.2	8.6	2341.75	0.8
**Kuwait**	4,310,108	1.0	5	46,140	−2.2	5.7	9.9	2908.25	0.8
**Oman**	4,644,384	1.5	3	21,540	1.3	4.3	10.5	1646.56	0.8
**Qatar**	2,716,391	0.8	2	70,070	4.2	2.8	8.3	2952.07	0.8
**Saudi Arabia**	36,947,025	1.5	3	28,690	−0.8	5.9	14.4	3029.48	0.8
**United Arab Emirates**	9,516,871	0.8	2	53,290	3.4	5.3	12.8	4065.61	0.9
**Upper-middle-income countries**									
**Iran**	89,172,767	0.7	8	4680	5.0	5.77	26.1	951.1	0.7
**Iraq**	45,504,560	2.2	3	5600	−2.9	5.25	6.9	513.2	0.6
**Jordan**	11,337,052	0.5	4	4460	2.6	7.29	7.9	738.5	0.7
**Lebanon**	5,353,930	−2.5	10	4410	−0.6	10.1	16.70%	528.0	0.7
**Libya**	6,888,388	1.1	5	7570	−1.7	4.0	6.42% (2011)	646.7	0.7
**Lower-middle-income countries**									
**Djibouti**	1,136,455	1.4	5	3450	6.7	2.8	4.2	150.3	0.5
**Egypt**	112,716,598	1.5	5	3900	3.8	4.6	6.8	615.4	0.7
**Morocco**	37,840,044	1.0	8	3700	3.2	5.7	7.1	515.7	0.6
**Pakistan**	240,485,658	2.0	4	1500	−0.0	2.9	4.5	167.3	0.5
**Sudan**	48,109,006	2.6	4	990	−12.0	2.8	7.8	127.3	0.5
**Tunisia**	12,458,223	0.8	9	3770	0.4	6.9	12.4	784.1	0.7
**Low-income countries**									
**Afghanistan**	42,239,854	2.7	2	360	−6.2	21.8	4.1	363.61	0.4
**Somalia**	18,143,378	3.1	3	610	3.1	N/A	N/A	N/A	0.3
**Syrian Arab Republic**	23,227,014	4.9	5	560	1.3	3.1	4.5	144.28	0.5
**Yemen**	34,449,825	2.2	3	820 (2018)	0.8	4.3	2.2	102.1	0.4

*Source:* The World Bank, World Population Review [5, 6].

LIC: Low-income country; LMIC: Lower-middle-income country; UMIC: Upper-middle-income country; GDP: Gross domestic product; GNI: Gross national income; HDI: Human Development Index.

## Health Outcomes and Epidemiological Transition

Health outcomes across the EMR show a pattern of overall progress, yet these gains are marked by deep disparities that align closely with the region’s economic stratification. Table 2 shows a clear gradient, where high-income countries report better health indicators than the low-income countries. This disparity is particularly striking in life expectancy, which reaches 79.7 years in high-income countries but falls to a low of 63.7 years in low-income countries. In 2021, the average life expectancy at birth in the EMR was approximately 68.5 years, with Qatar at the highest at 82 years and Somalia at the lowest at 56 years. The EMR has achieved notable reductions in neonatal, infant and under-five mortality rates from 1990 to 2022. In 1990, the neonatal mortality rate was 40 per 1000 live births, decreasing to 24.5 per 1000 live births by 2022 [7]. Similarly, the infant mortality rate decreased from 77 per 1000 live births in 1990 to 34.3 per 1000 live births in 2022 [8]. The under-five mortality rate decreased from 104.4 per 1000 live births in 1990 to 42.9 per 1000 live births in 2022 [9] (Table 2). Marked contrasts persist between high-income Gulf countries with relatively advanced healthcare infrastructure and lower-income or conflict-affected EMR countries, which experience fragile health systems and poorer health outcomes.

**Table 2:** Health indicators across EMR countries.

COUNTRY/REGION	LIFE EXPECTANCY (YEARS) (2021)	HEALTHY LIFE EXPECTANCY (YEARS) (2021)	NEONATAL MORTALITY (2022)	INFANT MORTALITY (2022)	<5 YEAR-OLD MORTALITY (2022)^a^	ADULT MORTALITY (2021)^b^	MATERNAL MORTALITY (2020)^c^
**Afghanistan**	63	50.4	36	45	58	344	620
**Bahrain**	79	64.2	3	6	7	95	15.9
**Djibouti**	63	57.0	29	44	52	246	234
**Egypt**	70	60.4	10	16	18	171	16.8
**Iran**	75	64.0	8	10	12	121	22
**Iraq**	71	61.0	13	20	24	148	76.1
**Jordan**	74	65.1	8	12	14	98	41.3
**Kuwait**	80	67.8	5	7	9	75	7.17
**Lebanon**	74	63.2	10	15	17	114	20.6
**Libya**	72	62.1	6	9	10	166	72.1
**Morocco**	75	62.3	11	15	17	136	71.9
**Occupied Palestinian Territory**	N/A	N/A	N/A	N/A	N/A	114	N/A
**Oman**	74	63.2	5	9	11	136	17
**Pakistan**	66	56.9	39	51	61	204	154
**Qatar**	82	66.2	3	5	5	71	7.61
**Saudi Arabia**	78	65.6	3	6	6	88	16.2
**Somalia**	56	47.4	35	68	106	405	621
**Sudan**	67.6	58.5	26	37	52	185	270
**Syrian Arab Republic**	72	62.3	11	18	21	131	29.9
**Tunisia**	74	63.6	8	10	12	115	36.6
**United Arab Emirates**	79	67.3	3	4	5	52	9.34
**Yemen**	64	56.5	22	33	41	226	183
**EMR**	68.5	59	24.5	34.3	42.9	173	-
**Global**	71.4	61.9	17.3	27.9	37.1	157	223.5
**Low-income countries**	63.7	55.3	26	46	65	245	409
**Lower-middle-income countries**	67.2	58.3	22	34	44	206	261
**Upper-middle-income countries**	74.2	65.1	7	11	13	129	62
**High-income countries**	79.7	68.1	3	4	5	107	12

Life expectancy (*Source*: WHO 2021).

Healthy life expectancy (*Source*: WHO till 2021).

Neonatal mortality rate (*Source*: WHO 2022).

Infant mortality rate and < 5-year-old mortality rate (*Source*: WHO 2022).

^a^ Per 1000 live births (*Source*: WHO till 2022)*.*

^b^ Per 1000 live births (*Source*: WHO till 2021)*.*

^c^ Per 100,000 live births (*Source*: WHO till 2020) [10].

The global neonatal mortality rate is estimated to be around 17.3 deaths per 1000 live births in 2022, improved by 53% from 36.8 deaths per 1000 live births in 1990 [11]. Similarly, the infant mortality rate in 2022 was 27.9 per 1000 live births compared to 63.7 per 1000 live births in 1990, improved by 56% [8]. Globally, the infant mortality rate has decreased from an estimated 32.7 deaths per 1000 live births in 2014 to 27.9 deaths per 1000 live births in 2022 (Figure 1a). The under-five mortality rate has improved over time from 53.3 in 2014 to 42.9 deaths per 1000 live births in 2022 in EMR. The global under-five mortality rate has witnessed improvement, decreasing from 44.3 per 1000 live births in 2014 to 37.1 per 1000 live births in 2021 (Figure 1b). The observed demographic and health disparities across EMR countries are reflected in the region’s evolving disease burden profile.

**Figure 1A:**
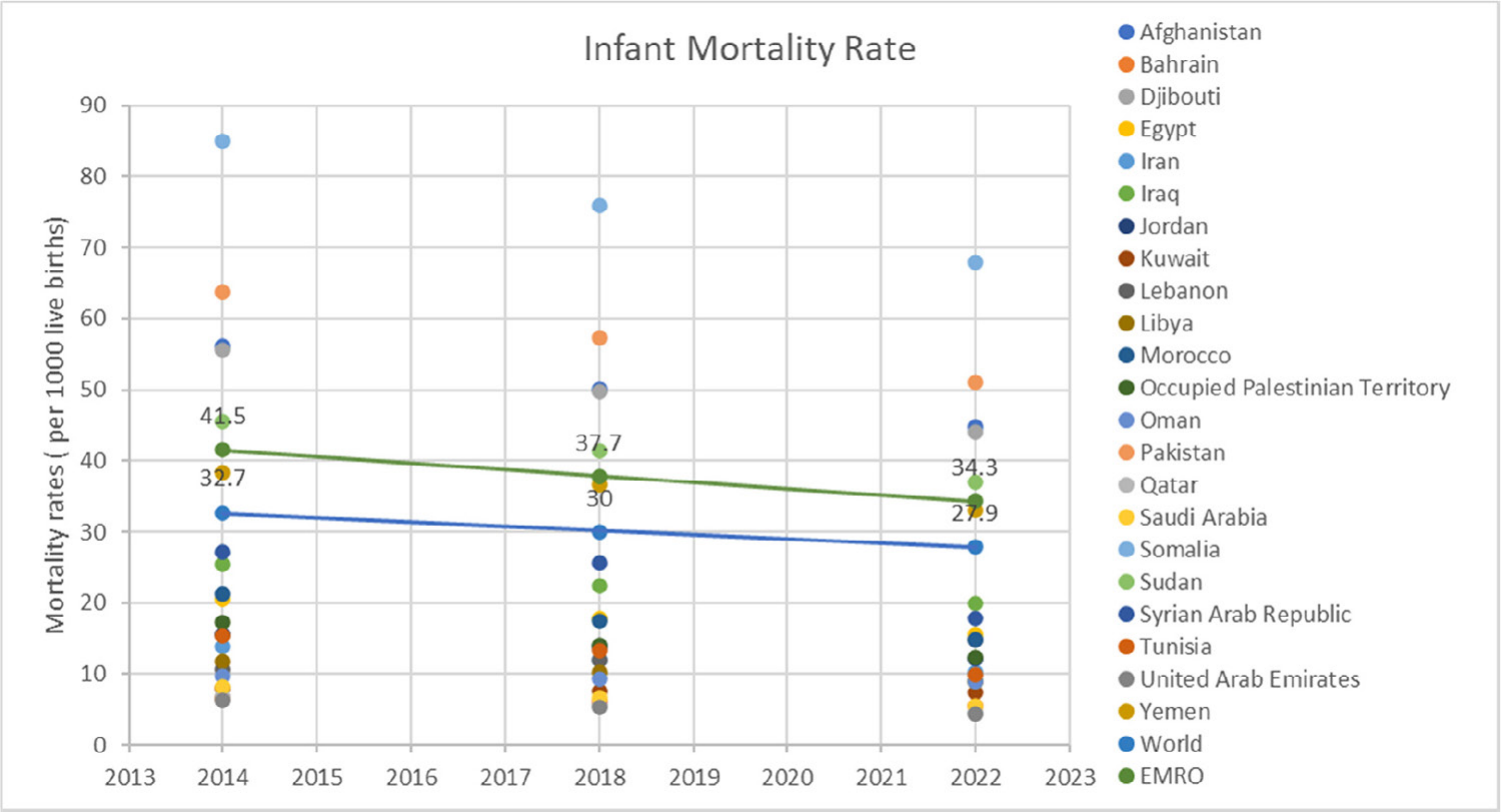
Trends in infant mortality rates in EMR countries, 2014–2022. Source: WHO [8].

**Figure 1B:**
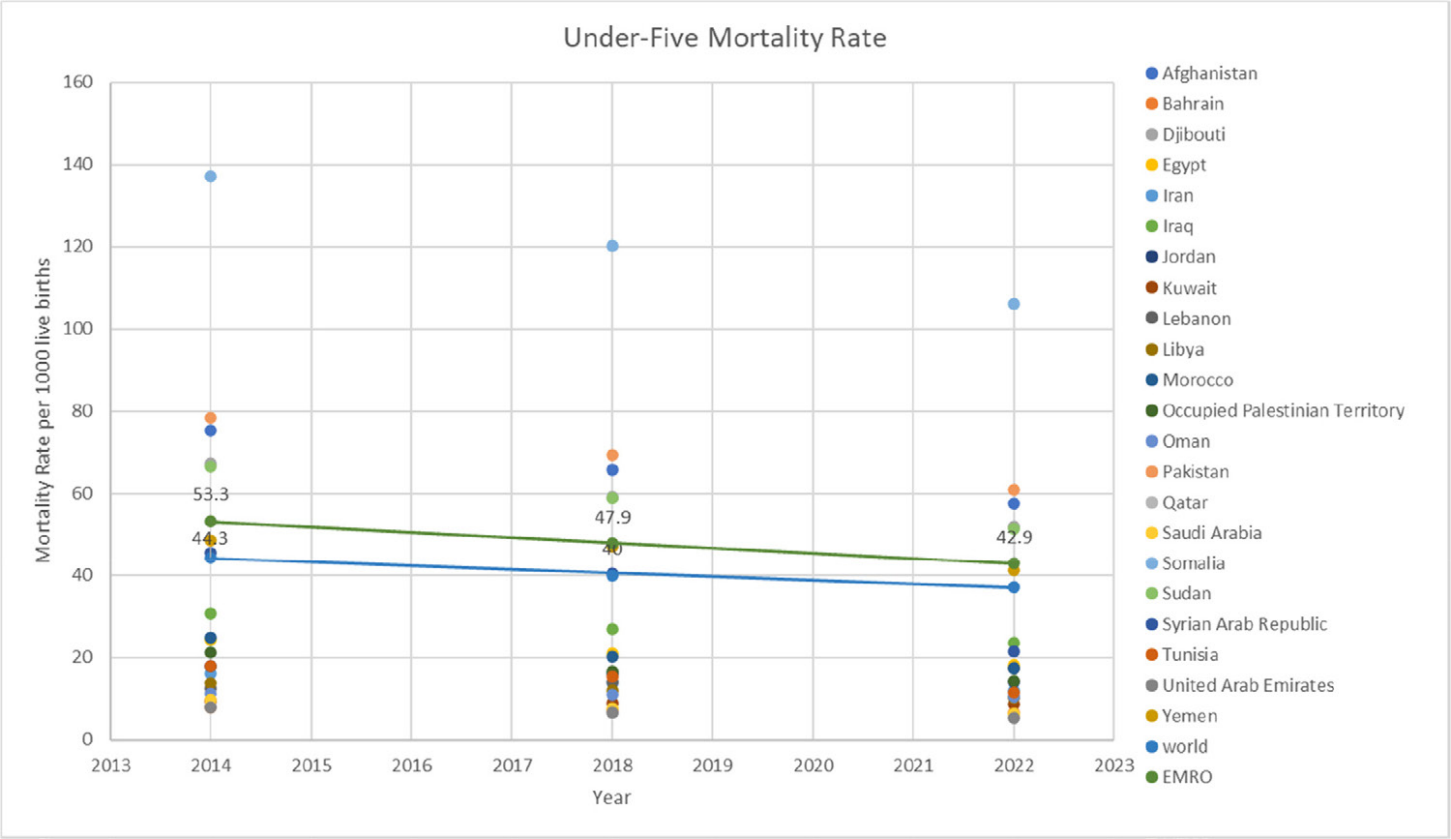
Trends in under-5 mortality rates in EMR countries, 2014–2022. Source: WHO [9]. These trends demonstrate gradual improvement in child survival indicators across the EMR over time; however, major disparities persist between high-income and conflict-affected countries, reflecting unequal healthcare access and health system capacity.

## The Triple Burden of Disease

**Figure 2:**
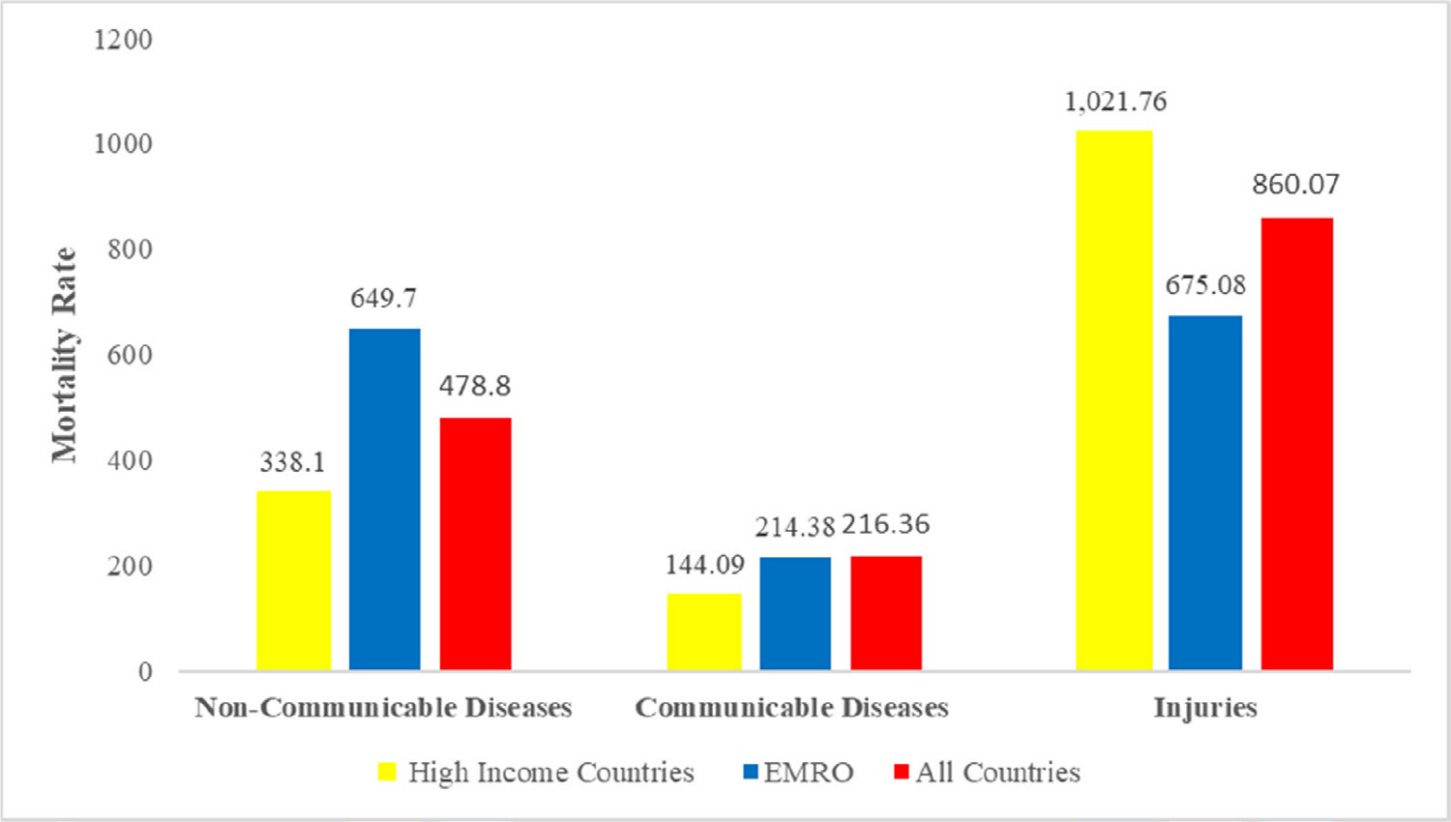
Mortality rates for NCDs, CDs and injuries in the EMR compared with global averages. Source: GBD 2021 [14], NCDs: WHO [13] 2021. *The substantially higher NCD mortality rates in the EMR compared with global averages highlight the accelerating epidemiological transition occurring across the region and the increasing pressure placed on healthcare systems*.

The disease burden in EMR is significant, encompassing CDs, NCDs and injuries. NCDs account for a substantial number of deaths in the EMR, with over half of these occurring prematurely (before the age of 70), which affects economically productive populations and places significant strain on health systems and national economies [12]. Figure 2 shows a high mortality rate of 649.7 from NCDs, exceeding both the rate in high-income countries (338.1) and the global average (478.8) [13, 14]. Cardiovascular diseases (CVDs) are the most prevalent cause of NCD deaths, followed by cancers, chronic respiratory diseases and diabetes, including kidney diseases caused by diabetes [12–15]. Mental health disorders, particularly depression and anxiety, are also highly prevalent. More than 150 million people are living with NCDs in the EMR [12]. In 2019, fatalities included approximately 1.46 million from CVDs, 431,000 from cancer and 186,000 from diabetes [15]. Additionally, NCDs are particularly concerning, accounting for over 2.2 million deaths in 2012, which represented 57% of total mortality in the region. The primary contributors to NCD-related deaths include CVDs (48%), cancers (16%), diabetes (5%) and chronic respiratory diseases (8%). If effective interventions are not implemented, projections suggest that NCD-related deaths could increase to 2.4 million by 2025 [16].

CDs also continue to cause significant challenges. The EMR’s mortality rate of 214.38 is considerably higher than in high-income countries (144.09) [13, 14]. In 2020, the EMR was heavily impacted by the COVID-19 pandemic, reporting around 5 million cases and 121,778 deaths, with a case-fatality ratio of 2.5% [17]. Additionally, the region experienced 10 major outbreaks of other emerging infectious diseases across seven countries. These included poliomyelitis in Afghanistan and Pakistan; chikungunya in Somalia and Sudan; cholera in Somalia and Yemen; dengue fever in Pakistan and Yemen; diphtheria in Sudan and Yemen; Crimean-Congo haemorrhagic fever in Afghanistan and Pakistan; chickenpox in Pakistan; Middle East respiratory syndrome (MERS) in Saudi Arabia; and viral haemorrhagic fever in Sudan. Collectively, these outbreaks resulted in over 430,000 reported cases and more than 400 deaths, which highlights ongoing public health challenges in controlling CDs [17]. An estimated 21 million individuals have chronic hepatitis B and 15 million have hepatitis C in the region, with Egypt and Pakistan accounting for over 80% of the hepatitis C burden. In 2017, approximately 771,000 people in the EMR developed tuberculosis (TB), which makes up 8% of the global burden, and 536,185 cases were notified, which indicates a treatment coverage rate of 68%. Five countries (Morocco, Pakistan, Somalia, Afghanistan and Sudan) bear 91% of the regional TB load. Drug-resistant TB also remains high, with an estimated 21,000 multidrug-resistant TB (MDR-TB) cases in 2017, among which only 4353 (21%) were initiated on treatment. Malaria elimination efforts are ongoing in Iran and Saudi Arabia; local cases in Iran decreased to 20 in 2018, whereas in Saudi Arabia, cases remained below 100 between 2010 and 2015 but rose to 194 in 2018 [18].

Injuries are a major health concern for children and adolescents in the EMR, which has some of the highest injury-related fatality rates globally. In 2017, the fatality rate for this demographic was 43.2 per 100,000, compared to a global average of 27.2 per 100,000. This highlights the urgent need for targeted interventions to reduce injury risks [19]. In 2019, the injury mortality rates for adults aged 50 and older in the EMR were 92.4 deaths per 100,000 for those aged 50–69, and 216.4 deaths per 100,000 for individuals 70 and older, both exceeding global averages [20]. Understanding the persistence of the triple burden requires examination of the broader determinants influencing health outcomes across the region. Collectively, these findings indicate that the EMR continues to experience an incomplete epidemiological transition, where CDs and injuries coexist with a rapidly increasing burden of NCDs. This overlapping burden places substantial pressure on already fragile health systems, particularly in conflict-affected and resource-limited settings.

## Underlying Determinants of Health

**Figure 3:**
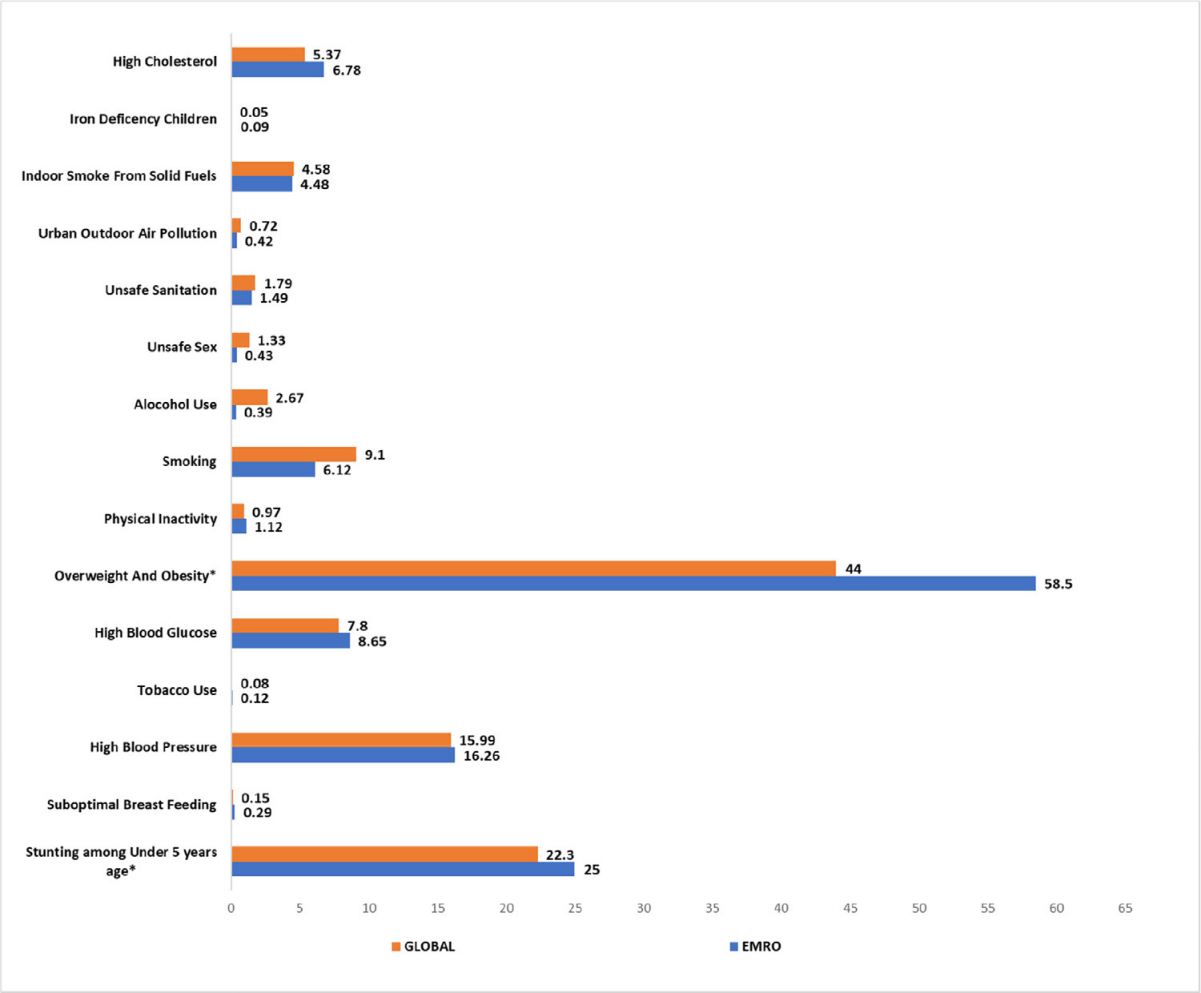
Prevalence of major risk factors in the EMR compared with global averages. * Prevalence of risk factors comparing EMR to the global averages (2022) [14].

The prevalence of specific risk factors associated with significant causes of morbidity and mortality burden in the EMR are high blood pressure (16.26%), high blood glucose (8.65%), smoking (6.12%), alcohol consumption (0.39%), high cholesterol (6.78%), low physical activity (1.12%), air pollution (0.42%), stunting among under 5 years (25%), suboptimal breastfeeding (0.29%), iron deficiency (0.09%), overweight and obesity (58.5%), tobacco use (0.12%), unsafe sex (0.43%), unsafe sanitation (1.49%) and indoor smoke from solid fuel (4.48%) as shown in Figure 3.

Overall, the health landscape of the EMR is significantly shaped by three principal determinants: socioeconomic factors, lifestyle and behavioural factors and environmental factors.

### Socioeconomic factors

Socioeconomic factors, including poverty, educational attainment and employment opportunities, are fundamental in determining individuals’ access to healthcare and their overall health outcomes. The EMR displays significant disparities in these areas, with marginalised populations encountering elevated health risks due to constrained resources and limited opportunities. Especially, individuals from lower socioeconomic backgrounds are often more likely to engage in unhealthy behaviours, thereby exacerbating health disparities [21]. The intersection of these determinants creates a complex web of health challenges, where issues like illiteracy and gender inequality further exacerbate the situation.

Socioeconomic factors deeply influence disease burden in the EMR. Lower-income countries experience a higher prevalence of NCDs like ischemic heart disease and diabetes, which have seen rising mortality rates. Access to quality healthcare is crucial; inadequate resources in low- and middle-income nations lead to poorer health outcomes [4]. Education and women’s empowerment also play significant roles, as higher educational levels are linked to better health choices [22]. Furthermore, urbanisation has resulted in more sedentary lifestyles and unhealthy eating habits. Addressing these socioeconomic determinants through strategies focused on improving income equality, healthcare access, education and social justice is vital to reducing the burden of disease in the EMR.

In the EMR, various risk factors contribute to the prevalence of infectious diseases. Socioeconomic challenges, such as poverty and limited access to healthcare, impede effective disease prevention and treatment, particularly in countries facing humanitarian crises and significant population displacement. It is essential to address these diverse risk factors to improve public health outcomes and manage infectious disease outbreaks in the EMR.

### Lifestyle and behavioural factors

Lifestyle choices, including dietary habits, physical inactivity, tobacco consumption and alcohol use, play a crucial role in determining health outcomes across the region. The EMR is undergoing a significant nutrition transition, marked by dietary patterns and lifestyle changes. Alarmingly, the region has high obesity rates, with approximately 53% of women, 45% of men and 8% of school-age children classified as obese. This trend is linked to an increase in chronic diseases, including CVDs, type 2 diabetes, and certain types of cancer [23]. As of 2014, approximately 50.1% of adult women and 43.8% of men in the region were classified as overweight or obese, reflecting a critical public health challenge exacerbated by epidemiological and nutritional transitions. Additionally, some countries in the EMR reported childhood obesity rates exceeding 15%, surpassing the global average of 7% [24]. In low- and middle-income countries within the EMR, the rates of adult obesity vary between 8.8% and 31.3%. These figures are approaching those observed in higher-income countries, such as the United States, where the prevalence of obesity is approximately 34.9%. Among adolescents, the prevalence of obesity ranges from 1% to 8.9%, which is significantly lower than the rate of about 20.5% reported in the United States [25, 26].

The prevalence of stunting among under 5 years in EMR stands at 25%, surpassing the global rate of 22.3%. The prevalence of stunting exhibits considerable variation across countries within the EMR. For example, Yemen reports one of the highest rates at 46.5%, closely followed by Afghanistan at 40.9%. In contrast, countries such as Kuwait and Iran demonstrate significantly lower rates of 4.9% and 6.8%, respectively [27]. In the EMR, about 19.31% of newborns are classified as having low birth weight (LBW), which is a significant public health issue. Additionally, anaemia prevalence varies from 7.4% to 88% in children under 5 and 19.9% to 63% in women of childbearing age [28].

Physical inactivity presents a pressing public health concern in the EMR, with alarming prevalence rates among adults and adolescents. The region reports one of the highest levels of physical inactivity globally, with approximately 43.2% of adults not engaging in adequate physical activity to meet established public health guidelines, a statistic closely mirroring that of the Americas, which stands at 43.3%. This significant level of inactivity has serious implications, contributing to a considerable burden of NCDs within the region. Specifically, it accounts for 8% of coronary heart disease cases, 10% of type 2 diabetes and 14% of both breast and colon cancers [29]. Among adolescents in the EMR, the situation is similarly concerning. Research indicates that only 19% of adolescents achieve the recommended levels of physical activity, whereas 29% demonstrate elevated levels of sedentary behaviour [30].

Tobacco consumption continues to pose a considerable public health challenge in the EMR, with prevalence rates varying significantly across different countries. The age-standardised prevalence of current tobacco smoking among individuals aged 15 years and older ranges from 8.1% in Oman to 35.0% in Jordan and Lebanon, thus underscoring the substantial regional disparities in tobacco consumption patterns [31].

The prevalence of elevated cholesterol levels within EMR constitutes a significant public health challenge, as it is intimately associated with the increasing incidence of CVDs. Current estimates suggest that the proportion of adults exhibiting elevated total cholesterol levels in the region ranges from 31% to 58%. This widespread concern contributes substantially to the overall risk of CVDs in countries within EMR [32]. Elevated cholesterol levels represent a significant risk factor for CVDs, which is responsible for approximately 34% of fatalities attributed to NCDs [33]. The prevalence of high cholesterol levels varies significantly across countries within the EMR. Notably, nations such as Bahrain and Kuwait exhibit particularly elevated rates of hypercholesterolemia, with research indicating that nearly 50% of the adult population in these countries may present with elevated cholesterol levels [34].

In the EMR, alcohol consumption constitutes a multifaceted public health challenge, marked by relatively low prevalence rates in comparison to global averages. Recent estimates reveal that over 30 million adults within EMR reported alcohol use in the past 12 months, resulting in an overall prevalence rate of 6.2% among the adult population. This figure signifies a considerable increase from the 2.9% reported by the WHO in 2016, indicating an emerging upward trend in alcohol consumption within the region [35].

### Environmental factors

Environmental factors, including pollution and urbanisation, significantly burden public health. It is estimated that environmental hazards are associated with approximately 22% of total regional mortality, disproportionately impacting vulnerable populations such as children and the elderly [36]. Environmental determinants substantially contribute to the burden of NCDs in the EMR.

Among these, air pollution stands out as a primary environmental risk, ranking immediately after high blood pressure and tobacco use as a leading cause of NCDs. Air pollution is responsible for a considerable number of fatalities resulting from conditions such as ischemic heart disease, stroke and lung cancer; approximately 85% of the 7 million deaths attributed to air pollution are linked to NCDs. Furthermore, climate change compounds health risks by increasing the frequency of extreme weather events and exacerbating food and water insecurity, thereby triggering and aggravating NCDs. Vulnerable populations, particularly individuals already affected by NCDs, experience disproportionately severe impacts from these changes. Additionally, exposure to toxic chemicals and heavy metals in the environment contributes to the prevalence of NCDs, as early life exposure can significantly elevate risk factors throughout a person’s lifetime [37].

Household air pollution (HAP) represents a significant risk factor for NCDs in the EMR. This issue predominantly arises from the use of solid fuels for cooking and heating, which emit harmful pollutants into residential spaces. The adverse health effects associated with these pollutants are well-documented, with links to respiratory diseases, cardiovascular conditions and various forms of cancer, thereby contributing to a considerable disease burden within the region. Notably, countries such as Afghanistan, Pakistan and Yemen exhibit elevated levels of HAP, which exacerbates existing health challenges and contributes to increased mortality rates from NCDs [38].

Environmental conditions, including poor sanitation and hygiene, elevate the risk of waterborne diseases like cholera and typhoid fever, especially in rapidly urbanising areas. Climate change exacerbates these risks by altering disease transmission patterns and increasing the frequency of extreme weather events that disrupt health services. Additionally, increased human–animal interactions due to agricultural practices facilitate the spread of zoonotic diseases.

The interaction between socioeconomic inequalities, behavioural risk factors and environmental exposures demonstrates that the burden of disease in the EMR cannot be addressed through disease-specific interventions alone. Rapid urbanisation, political instability, forced displacement and widening income inequalities have increased exposure to unhealthy lifestyles while also weakening access to preventive healthcare services. In conflict-affected countries, these vulnerabilities are compounded by poor sanitation, food insecurity, interrupted vaccination programmes, disrupted supply chains and fragile healthcare infrastructure. As a result, CDs, NCDs and injury-related risks are not separate challenges, but overlapping outcomes of deeper structural and environmental conditions. Addressing these determinants therefore requires integrated and multi-sectoral public health strategies that combine disease prevention, health system strengthening, social protection, environmental action and improved access to primary health care (PHC).

## Health System Capacities and Constraints

Healthcare systems across the EMR are characterised by high diversity and significant challenges. The region’s 22 countries face a combination of unsustainable financing, political instability, a dual disease burden and critical health workforce shortages, all of which affect the goal of universal health coverage (UHC) for its nearly 745 million people [39].

Inequitable healthcare financing is a primary concern. Many countries depend heavily on out-of-pocket (OOP) payments, which constitute an average of 40% of total health expenditure. This reliance on direct payments is the most inefficient funding model and pushes an estimated 55 million people in the region into poverty due to health costs [40, 41]. PHC systems remain under-resourced across many EMR countries. A 2022 WHO report noted that only half of EMR countries have a national PHC policy, and fewer still have dedicated budgets, leading to under-equipped facilities that cannot meet community needs [42]. This forces patients into more expensive hospital care and shifts focus away from essential prevention and health promotion. As a result, progress towards UHC is slow, with the region’s Service Coverage Index at just 58 out of 100 in 2019 [43].

A severe health workforce crisis exacerbates these issues, with a projected shortfall of 2.1 million health workers by 2030 [44]. The EMR has the second lowest density of nurses among all WHO regions, with 15.6 nurses per 10,000 population [45]. The variations in workforce and infrastructure across the region are detailed in Table 3. Adding to the pressure, efforts to combat NCDs are hindered by inadequate screening, low public awareness and a lack of surveillance data, particularly in low-income and conflict-affected countries. Finally, low health literacy across the region impedes disease prevention efforts, highlighting a critical need for effective, culturally appropriate health education programmes [46].

**Table 3:** Health workforce indicators in EMR countries.

COUNTRIES	PHYSICIANS^a^	NURSES^b^	COMMUNITY HEALTH WORKERS^b^	HOSPITAL BEDS^b^
**Afghanistan**	0.3 (2020)	4.5 (2018)	8.0 (2018)	3.6 (2021)
**Bahrain**	0.8 (2016)	23.4 (2016)	N/A	17.4 (2019)
**Djibouti**	0.2 (2014)	6.6 (2014)	N/A	14.2 (2018)
**Egypt**	0.7 (2019)	18.2 (2018)	0.09 (2018)	11.3 (2020)
**Iraq**	0.9 (2020)	19.8 (2018)	N/A	18.6 (2019)
**Iran**	1.5 (2018)	26.2 (2022)	3.5 (2016)	13 (2021)
**Jordan**	2.5 (2019)	31.6 (2019)	N/A	13.8 (2021)
**Kuwait**	2.3 (2020)	45.8 (2020)	N/A	23.5 (2020)
**Lebanon**	2.6 (2019)	19.2 (2018)	N/A	27.3 (2021)
**Libya**	2.2 (2017)	67.3 (2017)	N/A	32 (2021)
**Morocco**	0.7 (2017)	13.9 (2017)	0.2 (2020)	7.3 (2020)
**Oman**	2.0 (2020)	48.1 (2022)	N/A	11.6 (2020)
**Occupied Palestinian Territory**	N/A	19.5 (2018)	N/A	N/A
**Pakistan**	1.1 (2019)	4.6 (2019)	0.7 (2019)	5.1 (2019)
**Qatar**	2.5 (2018)	72.3 (2018)	N/A	11.2 (2019)
**Saudi Arabia**	2.8 (2021)	55.0 (2022)	1.5 (2018)	21.5 (2021)
**Somalia**	0.0 (2014)	1.1 (2014)	0.9 (2020)	8.7 (2019)
**Sudan**	0.3 (2017)	11.4 (2018)	1.5 (2004)	6.6 (2020)
**Syrian Arab Republic**	1.2 (2016)	14.1 (2016)	N/A	14.3 (2021)
**Tunisia**	1.3 (2017)	21.2 (2021)	N/A	24.2 (2021)
**United Arab Emirates**	2.9 (2020)	63.8 (2021)	N/A	19.8 (2021)
**Yemen**	0.3 (2014)	7.2 (2018)	0.01 (2010)	5.8 (2014)

Hospital beds (*Source*: WHO) [47].

Nurses (*Source*: WHO) [48].

Community health workers (*Source*: WHO) [49].

Physicians (*Source*: World Bank Group) [50].

^a^ Per 1000 population.

^b^ Per 10,000 population.

The United Nations and WHO regional framework for action on NCDs aims to guide countries in accelerating interventions to meet global 2025 and 2030 targets. However, NCD surveillance capacities vary between high-income states and low-income or conflict-affected countries, and a persistent lack of robust, localised data hinders the formulation of effective, evidence-based prevention and control policies across the region [46]. These systemic limitations undermine the region’s ability to effectively respond to both emerging infectious threats and the rising burden of chronic diseases. Strengthening PHC systems, workforce capacity, surveillance mechanisms and sustainable financing models will be essential for improving long-term health resilience in the EMR. Key regional priority gaps identified in this review include unequal healthcare access, insufficient PHC investment, limited workforce capacity, fragmented surveillance systems and persistent disparities between high-income and conflict-affected EMR countries.

## Opportunities for Regional Health Governance

The EMRO has demonstrated a commitment to advancing public health, yet significant challenges persist, which demand targeted efforts to achieve global, regional and national health goals. The region faces a complex health landscape, with a heavy burden of NCDs and CDs, the impacts of humanitarian crises and a struggle to strengthen health systems. Enhancing policy and legislative frameworks, promoting PHC, and ensuring UHC are fundamental to achieving progress. To meet the 2025 and 2030 NCD targets, it is crucial for EMR countries to increase the adoption and implementation of the guidance and tools outlined in the regional framework for action on the prevention and control of NCDs [51]. This will be crucial for improving access, coverage and quality of NCD prevention and control interventions across the region.

Addressing NCDs and achieving UHC in the EMRO region depends on three core elements: promoting the capacity of health systems, confronting the inequalities in the NCD burden and mitigating the economic impacts inflicted by these diseases. The delivery of integrated NCD services at the PHC level, supported by evidence-based guidelines, essential technologies, a skilled health workforce and robust health information systems, is of great importance for the region.

UHC is a cornerstone of the WHO’s vision for the EMR region. While the UHC service coverage index for the region stood at 58 (out of 100) in 2019, which is below the global average of 66 (out of 100) [43], significant disparities persist between and within countries. High OOP health expenditures continue to be a major barrier to accessing care and push millions into poverty. It is crucial to formulate and implement robust health policies and financing strategies that support populations while promoting equitable and efficient resource allocation. As nations in the EMR region strive towards UHC, establishing strong mechanisms for measurement, monitoring and accountability is critical. This needs a shift towards enhancing frontline services, focusing on health system performance and outcomes and promoting a ‘Health in All Policies’ strategy by governments. Key areas for action include maintaining high-quality care, integrating health services, leveraging new technologies, promoting community engagement, regulating private providers, strengthening outbreak surveillance and ensuring health equity.

To advance towards UHC, an alignment between Public Financial Management (PFM) and health financing objectives is essential. PFM systems must be geared to support national health goals, requiring a renewed commitment and sustained collaboration between ministries of health and finance. Health ministries across the EMR region should advocate for health-sector-specific needs, such as greater flexibility in financial management and enhanced accountability of health facilities within the broader PFM reform discourse. Collaborative efforts between finance and health officials are vital to optimise resource allocation in the health sector, with a focus on goal-oriented budgeting, multi-year planning and improved forecasting.

Fragmented healthcare systems cause a substantial challenge in the EMR region, as they do globally. A key strategy to address this is the move towards PHC-oriented models of care that provide UHC without financial barriers and invest in high-value services for all. Reducing administrative burdens that detract from health improvement efforts and investing in social services, particularly for vulnerable populations, are also crucial. A unified and integrated health system can yield significant benefits for the EMR region, including improved administrative efficiency and equitable access to care. While a single model may not be universally applicable, the experiences of countries within the region that are reforming their health systems to strengthen PHC and introduce prepayment schemes, such as health insurance, can offer valuable lessons. To tackle the rising issue of chronic diseases, rapid urbanisation and fragmented care, an integrated and person-centered approach to care is necessary across all levels. Enhancing the quality and safety of healthcare services is also fundamental to meeting public expectations and rebuilding trust in health systems.

Despite several WHO EMRO strategies and regional policy frameworks, implementation gaps remain substantial across many EMR countries. Political instability, weak governance, fragmented financing and donor dependence continue to limit sustainable reform, especially in low-income and conflict-affected settings. Stronger accountability, domestic investment in PHC, improved surveillance and better coordination between health and finance sectors are needed to translate regional strategies into measurable progress.

Addressing the social determinants of health is fundamental to achieving health equity and social justice, particularly in the low- and middle-income countries of the EMR region. The World Health Assembly resolution on the social determinants of health highlights the importance of measuring health inequities, integrating social determinants into public health programmes, adopting a holistic, multi-sectoral governmental approach and aligning these efforts with the renewal of PHC. To support evidence-based decision-making for disease prevention and control, the region’s health information and surveillance systems must be significantly improved. The recently launched regional strategy to enhance and digitalise health information systems provides a roadmap for generating high-quality, timely and reliable data. Overcoming the challenges of fragmented and often paper-based systems requires a concerted effort from policymakers, public health professionals, researchers and local stakeholders to take decisive action and promote a culture of data-driven policy-making.

## Conclusion

The EMR faces a complex and evolving public health burden shaped by socioeconomic inequalities, conflict, displacement, environmental risks and persistent health system constraints. Although progress has been made in several health indicators, major disparities remain between high-income countries and low-income or conflict-affected settings. The continuing overlap of NCDs, CDs, and injuries places substantial pressure on already stretched health systems.

Addressing these challenges requires stronger investment in PHC, preventive services, health workforce development, surveillance systems and sustainable health financing. Policy efforts should also focus on reducing health inequalities, strengthening health information systems and adopting multi-sectoral approaches that address the wider social, behavioural and environmental determinants of health. Coordinated regional action will be essential to improve health outcomes, advance UHC and build more resilient health systems across the EMR.

## Limitations

This review has some limitations. It is based on secondary data from international databases, institutional reports and published literature, and the availability, completeness and quality of these data vary across EMR countries. These limitations are likely to be greater in conflict-affected settings, where health information systems and surveillance mechanisms may be disrupted.

As this was a descriptive narrative review, it did not involve a systematic search strategy, formal quality appraisal, primary data collection, statistical modelling or meta-analysis. Therefore, the findings should be interpreted as a broad regional overview rather than as a causal or quantitative synthesis. Despite these limitations, the review provides a useful summary of major epidemiological challenges, health disparities and health system priorities in the EMR.
